# Adaptation to Brazilian Portuguese and Latin-American Spanish and psychometric properties of the Mental Illness Clinicians’ Attitudes Scale (MICA v4)

**DOI:** 10.47626/2237-6089-2021-0291

**Published:** 2023-03-07

**Authors:** Angel O. Rojas Vistorte, Wagner Ribeiro, Carolina Ziebold, Elson Asevedo, Sara Evans-Lacko, Denisse Jaen Varas, Nataly Gutierrez, Michel Haddad, Oscar Ulloa, Ricel Martínez, Andresa Sartor Harada, Jair de Jesus Mari

**Affiliations:** 1 Departamento de Psiquiatria Universidade Federal de São Paulo São Paulo SP Brazil Departamento de Psiquiatria, Universidade Federal de São Paulo (UNIFESP), São Paulo, SP, Brazil.; 2 London School of Economics and Political Science London United Kingdom Personal Social Services Research Unit, London School of Economics and Political Science, London, United Kingdom.; 3 Global Mental Health Program Columbia University New York NY USA Global Mental Health Program, Columbia University, New York, NY, USA.; 4 Universidad Internacional de La Rioja Logrono La Rioja Spain Universidad Internacional de La Rioja, Logrono, La Rioja, Spain.; 5 Institute of Psychiatry, Psychology & Neuroscience Health Service and Population Research Department King’s College London London United Kingdom Institute of Psychiatry, Psychology & Neuroscience, Health Service and Population Research Department, King’s College London, London, United Kingdom.

**Keywords:** Attitude, primary care physicians, stigma, psychometric validation, measure

## Abstract

**Objective:**

To describe translation to Spanish and Portuguese and adaptation of the Mental Illness Clinicians’ Attitudes Scale version 4 (MICA v4).

**Methods:**

The questionnaire was administered to primary care physicians (PCPs) from four Latin-American countries, Brazil, Bolivia, Chile, and Cuba. The validation process included four phases: 1) translation of the questionnaire to Spanish and Portuguese; 2) assessment of face validity; 3) assessment of reliability; and 4) evaluation of construct validity with confirmatory factor analysis (CFA).

**Results:**

The study sample comprised 427 PCPs. The mean age of the Spanish-speaking sample (n = 252) was 40.1 (S.D = 9.7) years and the mean age of the Portuguese-speaking sample (n = 150) was 40.2 (S.D = 10.9) years. Both models demonstrated “appropriate” internal reliability. Total omega was 0.91 for the Spanish-speaking sample and 0.89 for the Portuguese-speaking sample. The CFA of both questionnaires showed an appropriate fit for a three-factor model (Portuguese: CFI = 0.927; TLI = 0.913; RMSEA = 0.066; Spanish: CFI = 0.945; TLI = 0.935; RMSEA = 0.068).

**Conclusion:**

The Latin-American versions of the MICA v4 in Spanish and Brazilian Portuguese have appropriate psychometric properties, good internal consistency, and are applicable to and acceptable in the Latin-American context. The instrument proved its validity for collecting data on stigmatizing attitudes among health professionals in different contexts and cultures.

## Introduction

Mental disorders account for more than 13% of the global burden of diseases and have a lifetime prevalence among adults ranging from 12.2 to 48.6%, and 12-month prevalence ranging from 8.4 to 29.1%.^1 , 2^ Primary health care services (PHCS) are the main settings in which people with mental health disorders are treated. Evidence shows that non-specialist professionals provide more than 90% of mental health care worldwide.^3 , 4^

Despite programs to train primary care professionals to treat mental health problems, access to treatment is still low among people with mental disorders.^5^ Only 16.5% of people with major depression receive minimally adequate treatment (22.4%, 11.4%, and 3.7% in high, upper middle, and low/lower middle-income countries respectively).^6^ Beyond scarcity of economic resources, one of the main obstacles that impedes access to mental health treatment is stigmatizing attitudes toward people with mental health problems.^7^

Stigma is defined as a process involving labeling, separation, stereotype endorsement, prejudice, and discrimination, in a context in which social, economic, or political power is exercised to the detriment of members of the social group.^8^ It comprises three related dimensions, which, when combined, are powerful drivers of social exclusion: a) lack of knowledge (ignorance and misinformation); b) negative attitudes (prejudice); and c) excluding or avoiding behaviors (discrimination).^9 - 13^ Four types of stigma toward people with mental illness are common: a) social or public stigma; b) structural stigma; c) family stigma; and d) self or internalized stigma.^14 - 16^ All these types of stigma are associated with increased functional impairment among those who experience stigma and discrimination,^17^ affecting their health, social inclusion, and quality of life.^7 , 18 - 20^

Considering the consequences of stigma toward people with mental illness, the World Health Organization (WHO)^21^ has included the reduction of stigma, discrimination, and human rights violations through implementation of community mental health care as important goals in its Global Mental Health Action Plan (mhGAP) 2013-2020. Although the WHO recommends that mental disorders should be treated in primary care, some research suggests that health professionals manifest more negative ratings toward people with mental health than the general public.^1 , 2 , 4^

Studies carried out in Latin America have found high levels of public and family stigma toward individuals affected by mental illness were associated with high levels of functional impairment.^17^ However, little is known about stigmatizing attitudes toward mental health illness among health professionals in Latin American countries. Standardized instruments tailored for clinicians are not available and studies carried out in Latin American tend to use heterogeneous and unvalidated measures, which were mainly developed ad hoc, for example, in the context of evaluating a depression training program for clinicians. Consequently, it is difficult to compare/combine findings and to precisely assess and estimate the impact of stigma among health professionals in Latin America.^17 , 22 , 23^

Understanding health professionals’ attitudes is the first step toward evaluation of presence of stigma and proposing interventions to promote behavioral change. To assess attitudes towards mental illness among students or staff of any health care discipline, the English version of the Mental Illness Clinicians’ Attitude Scale (MICA v4)^24^ was translated and adapted for the Latin American context. Notwithstanding the important differences in each country in terms of contexts, structural installations, quantity of professionals, medications used, and policies regarding the treatment of mental disorders, which may influence stigmatizing attitudes toward mental health illness, adaptation of the MICA v4 scale to Portuguese and Spanish would provide researchers in Latin America with a valid, standardized, and feasible tool to assess stigma among primary care physicians (PCPs), who are the main professionals diagnosing and treating mental health problems.^25^

Thus, the aims of this study were to translate and adapt the MICA v4 to Brazilian Portuguese and Latin American Spanish and to assess its psychometric properties in a sample of PCPs from four Latin American countries.

## Methods

### Sample and procedures

We invited 550 PCPs from Bolivia (La Paz), Brazil (whole country), Cuba (in Brazil on the Mais Medicos program), and Chile (Santiago) to participate in the study. Data collection was carried out between April and July 2016, through an online survey using the Qualtrics platform.^26^ This is an online platform that enables administration of several questionnaires with a simple format for the participant. The PCP participants from Bolivia, Brazil, and Chile lived and worked in their respective countries. In contrast, the Cuban professionals were part of the Mais Medicos program and were working in different regions of Brazil. We obtained a list of Brazilian and Cuban PCPs from the health ministry of Brazil and of Bolivian and Chilean PCPs from the members of our team. As a criterion for inclusion, the first question on the questionnaire after agreeing to participate, asked whether the PCPs attended patients in primary care. Considering that patients with common mental disorders are supposed to be treated in primary care settings in all four of these countries, physicians who work in such settings are likely to encounter patients with common mental disorders in their clinical practice. Treating mental health patients was not a requirement, but the questionnaire does ask whether they see this type of patient.

### Instrument description

The MICA v4 is a 16-item scale developed by Gabbidon et al.,^24^ designed to assess medical students’ attitudes towards people with mental illness. Each item requires a response on a 6-point Likert scale (strongly agree, agree, somewhat agree, somewhat disagree, disagree, or strongly disagree). A single overall score is calculated by summing all individual items. Higher overall scores indicate more negative stigmatizing attitudes. The total score ranges from 16 to 96.^24^ The English version of the MICA v4 is shown in supplementary material S1 (online only).

### Translation of the MICA v4

The translation process followed the procedures proposed by Sartorius for translation of instruments designed to collect Health Measures in different cultural contexts.^27^ After approval had been granted by the author of the original scale,^24^ a panel of bilinguals (English-Spanish or English-Portuguese) was convened, made up of two Psychologists (AORV, CZ), three Psychiatrists (JJM, EA, DJV) and one General Practitioner (NG), who were native speakers of Spanish (AORV, CZ, DJV, NG) and Portuguese (JJM, EA).

Two independent translators who were not part of the study team translated the scale into each target language. The translated versions were then revised by a bilingual panel of Cuban (AORV), Bolivian (DJV, NG), and Chilean (CZ) specialists (Spanish version) and a bilingual panel of Brazilian specialists (JJ, EA), to check the conceptual, semantic, and technical equivalence between the translated versions and the original scale. Reviewers independently rated the quality of each translation and identified consistent differences between original and target versions. From this review, new versions were proposed and were then back-translated by an independent translator, for adjustment of the final Spanish and Portuguese versions, presented in Supplementary Materials S2 and S3, respectively (online only).

### Data and psychometric analysis

The Statistical Package for the Social Science 20.0 (SPSS)^28^ was used for the descriptive analyses. The Mplus software package, version 7.4,^29^ was used for analysis of psychometric properties. Initial analyses were carried out to test the normal distribution and assess the sample mean and standard deviation of the MICA v4 responses and to report differences in scores across the sociodemographic groups. The psychometric properties of the MICA v4 were established by assessing its face validity, reliability, and acceptability.

### Psychometric analysis

Confirmatory factor analysis (CFA) was performed for each language version to assess their construct validity by testing the goodness of fit of theoretical factor models to the data. The robust weighted least squares (WLSMV) parameter was used, as suggested for complex modeling using categorical variables. First we tested a model with all of the five factors found by the authors of the original MICA.^24^ This resulted in an unidentified model in our study, so we proposed an alternative theoretical model, with three sets of items loading onto three factors, which we labeled as “views of health and social care fields and mental illness,” “disclosure and knowledge of mental illness,” and “distinguishing mental/physical health.” The fit indices of the Mica v4 were analyzed using the following reference indices: the chi-squared test of model fit, which should not identify statistical significance (p > 0.05), the root mean square error of approximation, which should be close to or less than 0.8 (RMSEA < 0.08), and the Comparative fix index (CFI) and the Tucker-Lewis index (TLI), which should both be close to or greater than 0.9.^30^

### Face validity

The alpha versions of the questionnaire in Latin American Spanish and Brazilian Portuguese were administered to two groups of PCPs in Cuba and Brazil (50 professionals in each country) in the cities of Havana and São Paulo. Forty-seven professionals in Cuba and forty-six in Brazil completed the questionnaire. The professionals were asked to rate clarity and comprehensibility of each item and to provide suggestions on how to improve items that were unclear.

In the Spanish-speaking sample, items 2, 3, 4, 7, 8, 10, 11, 12, 13, 14, 15, and 16 were considered fully comprehensible by 100% of respondents (n = 47). Items 1, 5, 6, and 9 were considered comprehensible by 90% (n = 42), 86% (n = 40), 93% (n = 43), and 91% (n = 42) respectively. In the Portuguese-speaking sample, items 1, 2, 3, 5, 6, 7, 8, 10, 11, 12, 13, 14, 15, and 16 were considered fully comprehensible by 100% of respondents (n = 46). Items 4 and 9 were considered comprehensible by 91% (n = 41) and 87% (n = 40) respectively. These items are described in supplementary material S1. The alpha versions were slightly modified based on participants’ comments and sent to the panel of translators for analysis. The final versions were then administered to a sample of PCPs from Bolivia, Chile, Brazil, and Cuba.

### Reliability

The total omega (ω_t_) coefficient was computed to evaluate the reliability of the MICA v4. The ω_t_ coefficient can vary from 0 to 1, where higher scores indicate greater reliability.^31 , 32^ The omega has several advantages over alpha, such as: a) omega makes fewer and more realistic assumptions than alpha; b) problems associated with inflation and attenuation of internal consistency estimation are far less likely; c) employing ‘omega if item deleted’ in a sample is more likely to reflect the true population estimates of reliability through removal of a certain scale item; and d) calculation of omega alongside a confidence interval more closely reflects the variability in the estimation process, providing a more accurate degree of confidence in the consistency of administration of a scale.^33^ However, since Cronbach’s alpha was used in the original study, but we preferred to use omega, we also calculated Cronbach’s alpha in order to maintain comparability between the studies.

### Ethics statement

The study design and informed consent forms were reviewed and approved by the Ethics Committee at the Universidade Federal de São Paulo (UNIFESP; CEP Project n1496/2015, number 51492815.5.0000.5505). Participants were given detailed information about the research project and were enrolled at liberty to choose to participate in the study or not. To ensure anonymity, participants were de-identified in the database.

## Results

All the steps of the translation process were followed as planned and final Latin American versions of the scale were produced in Spanish and Brazilian Portuguese. No significant adaptations were made to the Brazilian Portuguese version. In the Spanish version, “would not bother” was initially translated as “no me importaría”; but in accordance with participants’ suggestions, “no me importaría” was replaced with “no me incomodaría,” which was back-translated as “wouldn’t mind.” Additionally, the original version uses the phrase “a severe mental illness,” which was translated literally as “severa,” but in accordance with participants’ suggestions we decided to use “grave.”

### Sample characteristics

The questionnaire was sent to 550 PCPs from the four countries between April and July of 2016, and was completed by 427 professionals (77.6% response rate), 252 from Spanish-speaking countries and 150 from Brazil. The sample comprised 255 females and 172 males. The distributions of social and demographic characteristics of the PCPs in the four countries can be seen in Table 1 . The mean age of the Spanish-speaking sample was 40.1 (S.D = 9.7) with a range of 25 to 66 years. The mean age of the Portuguese-speaking sample was 40.2 (S.D = 10.9) with a range of 26 to 72 years. There were statistically significant differences between the four countries for age (p = 0.02), years of training (p < 0.01), and years of experience (p < 0.01), with the Brazilian sample having the highest percentage of physicians over the age of 41 years and the longest duration of training and experience.

**Table 1:** Distribution of social and demographic characteristics of the primary care physicians, by country

Items	Portuguese	Spanish			*χ^2^* test p values

	Brazil (n = 150)	Bolivia (n = 38)	Chile (n = 32)	Cuba (n = 192)	
Gender					
Female	97 (65.1)	19 (50)	21 (65.6)	108 (61.5)	0.23
Male	53 (34.9)	19 (50)	11 (34.4)	74 (38.5)	
Age					
≥ 41	85 (56.8)	12 (31.5)	18 (56.3)	87 (45.3)	0.03^†^
< 41	65 (43.2)	26 (68.5)	14 (43.7)	105 (54.7)	
Training years					
≥ 11.5	112 (75.3)	24 (63.1)	10 (31.2)	91 (47.4)	0.001^‡^
< 11.5	38 (24.7)	14 (36.9)	22 (68.8)	101 (52.6)	
Exp years*					
≥ 11.9	92 (61.6)	25 (65.8)	19 (59.4)	85 (44.3)	0.003§
< 11.9	58 (38.4)	13 (34.2)	13 (40.6)	107 (55.7)	

Data presented as n (%).

* Years of experience.

^†^ Chi-square test = 9.11, degrees of freedom = 3, pairwise comparisons showed that the Brazilian sample was older than the Bolivian participants.

^‡^ Chi-square test = 30.7, degrees of freedom = 3, pairwise comparisons showed that the Cuban sample had fewer years of training compared with the Brazilian and Bolivians participants.

^**§**^ Chi-square test = 13.8, degrees of freedom = 3, pairwise comparisons showed that the Cuban sample had fewer years of experience working in primary health care compared to the Brazilian participants.

### Distribution of participant responses

The distribution of MICA responses, comparing the Portuguese and Spanish-speaking samples, can be found in Supplementary Material S4, available online-only. Among Spanish speakers, there were no significant between-countries differences. Between Portuguese and Spanish speakers, there were significant differences in five items: 5 (p = 0.03), 7 (p < 0.01), 11 (p = 0.01), 13 (p = 0.02) and 16 (p = 0.01).

### Internal consistency

The two proposed models had adequate internal reliability, the total omega coefficients for the MICA v4 were 0.91 for the Spanish-speaking sample and 0.89 for the Portuguese-speaking sample.^31 , 32^

### Confirmatory factor analysis and model fit

Initially, we tested a model with the five-factor solution proposed by the London study,^20^ resulting in an unidentified model. We therefore present an alternative model with three factors being as follows: a “views of health social care field and mental illness” factor, including seven items (3, 6, 9, 10, 11, 12, and 16); a “disclosure and knowledge of mental illness” factor, including four items (2, 4, 5, and 7); and a “distinguishing mental/physical health” factor, including five items (1, 8, 13, 14, and15).

This model had a good fit to the data, with factor loadings ranging from -0.123 to 0.957 in the Spanish-speaking sample, with item 3 “working in the mental health field is just as respectable as others fields of health and social care,” exhibiting the highest factor loading; and loadings from 0.201 to 0.883 in the Portuguese-speaking sample, with item 8 “being a health/social care professional in the area of mental health is not like being a real health/social care professional,” exhibiting the highest factor loading. The results of the CFA are shown in Table 2 . The fit indices for the Portuguese scale were: *χ* ^2^ = 166.67, degrees of freedom (df) = 101, p = 0.01; CFI = 0.927 (CFI < 0.90); TLI = 0.913 (TLI < 0.90) and RMSEA = 0.066 (RMSEA < 0.08) and indices for the Spanish scale were: *χ* ^2^ = 219.723, df = 101, p = 0.01; CFI = 0.945 (CFI < 0.90); TLI = 0.935 (TLI < 0.90) and RMSEA = 0.068 (RMSEA < 0.08).

**Table 2:** Confirmatory factors analysis: model fit of each version*

	Spanish	Portuguese
N	252	150
Comparative fix index	0.945	0.927
Tucker-Lewis index	0.935	0.913
Root mean square error of approximation	0.068	0.066
*χ^2^*	*χ^2^* = 166.67, df = 101	*χ^2^* = 219.723, df = 101

Spanish: p = 0.01; Portuguese: p = 0.05.

df = degrees of freedom.

* A model with three factors is proposed: 1) “Views of health social care and mental illness”; 2) “Disclosure and knowledge of mental illness”; and 3) “Distinguishing mental/physical health.” These reference indices were used: chi-squared test of model fit (p > 0.05), root mean square error of approximation < 0.08, comparative fix index > 0.90, and Tucker-Lewis index > 0.90.

## Discussion

The versions of the MICA v4 adapted for Latin American Spanish and Brazilian Portuguese were comprehensible and had good reliability in a sample of Latin American PCPs from four countries (Bolivia, Brazil, Chile, and Cuba). To the best of our knowledge, this is the first study conducting translation of MICA v4 into languages other than English followed by validation. Frequently, results of studies on stigma carried out in Latin America are limited by use of instruments that have not undergone adequate cross-cultural adaptation, leading to a biased interpretation of findings.

Our study included two different samples in Latin America – one from three Spanish-speaking countries (Bolivia, Chile, and Cuba), and one from a Portuguese-speaking country (Brazil). Therefore, the Portuguese-speaking sample was more homogeneous than the Spanish-speaking one. The Brazilian sample was older, with longer duration of training and more experience dealing with mental health problems in primary care.

The study that validated the original version of the MICA^24^ found five dimensions in the principal component analysis, but did not show good fit or was unidentified. In contrast, we propose a three-factor solution, which was found to have good adequate internal structure and good fit in our CFA, for both the Spanish and the Brazilian Portuguese versions.

The mean loading factors of our two samples (0.600 for the Portuguese-speaking sample and 0.620 in the Spanish-speaking sample) were similar to the mean loading factor in the original study (0.641). We got similar results in the internal consistency using Cronbach’s alpha, since in the original study they obtained (α = 0.79), while our two samples obtained α = 0.76 (Spanish-speaking sample) and α = 0.72 (Portuguese-speaking sample). In addition to alpha, we preferred to use ω_t_. These results were ω_t _= 0.89 for the Portuguese version, and ω_t _= 0.91 for the Spanish version, which are considered satisfactory. Although these results are considered good for a model with three factors, it is necessary to evaluate other theoretical models in order to obtain better results for the fitted model.

Understanding health professionals’ attitudes is the first step toward evaluating the presence of stigma and proposing interventions to promote behavioral change. Adaptation of the MICA v4 scale for Portuguese and Spanish provides researchers in Latin America with a valid, standardized, and feasible tool to assess stigma among PCPs, who are the main professionals diagnosing and treating mental health problems.

Many studies have demonstrated that lower levels of knowledge, lack of adequate training, and stigmatizing attitudes among PCPs were associated with poorer diagnostic and treatment efficacy in mental disorders.^34 , 35^

The objective of the MICA V4 is to assess attitudes towards mental illness among students or staff of any health care discipline. So, if we can identify or measure stigmatizing attitudes in PCPs, that could contribute to reducing the disparities in care of individuals with mental disorders,^36 - 39^ through programs or interventions, and reduce barriers to treatment faced by people with mental disorders, thereby increasing their access to optimal care, which would lead to improvement in their mental health status and well-being.

These interventions should consider the context of the primary care setting and consider barriers to treatment including lack of adequate time, training, competing agendas, and lack of adequate reimbursement. Ineffective treatment leads to patient and physician frustration because of lack of progress.^38^

The main limitation of this study is that we did not assess other psychometric properties of the scale such as concurrent validity, convergent/divergent validity, and test-retest reliability. Another limitation is that our samples may not be representative of Brazilian and Latin-American physicians because they only included professionals who had access to the internet and a small number of PCPs. At the same time differing cultural characteristics, structural installations, quantity of professionals, medications used, and policies regarding the treatment of mental disorders may influence stigmatizing attitudes toward mental health illness. Therefore, one should be cautious when generalizing our results to Latin America.

Despite these limitations, our study shows that the Latin-American versions of the MICA v4 in Spanish and Brazilian Portuguese have good internal consistency and good psychometric properties and that they are applicable to and acceptable in the Latin American context. Having versions of the MICA v4 in Spanish and Portuguese is an important step to advance understanding of health professionals’ attitudes towards mental illness in different contexts and languages, offering the opportunity to determine the magnitude of the stigma in this region and to develop intervention strategies to promote behavioral changes towards stigma.
