# Supplementary material for: Adaptation to Brazilian Portuguese and Latin-American Spanish and psychometric properties of the Mental Illness Clinicians’ Attitudes Scale (MICA v4)
Source: Trends Psychiatry Psychother. 2023 Mar 7;45:e20210291. doi: 10.47626/2237-6089-2021-0291 (PMC10164403; doi:10.47626/2237-6089-2021-0291)
Supplement: Supplementary file 1 [file 2238-0019-trends-45-e20210291-suppl1.pdf]

http://doi.org/10.47626/2237-6089-2021-0291

Supplementary Material S1

Page 1

Mental Illness: Clinicians' Attitudes Scale **MICA-4**

Note to researchers distributing this scale: please only use after reading instructions in "Manual for Researchers".

Instructions: for each of questions 1-16, please respond by **ticking one box only**. Mental illness here refers to conditions for which an individual would be seen by a psychiatrist.

		Strongly agree	Agree	Somewhat agree	Somewhat disagree	Disagree	Strongly disagree
1	I just learn about mental health when I have to, and would not bother reading additional material on it.						
2	People with a severe mental illness can never recover enough to have a good quality of life.						
3	Working in the mental health field is just as respectable as other fields of health and social care.						
4	If I had a mental illness, I would never admit this to my **friends** because I would fear being treated differently.						
5	People with a severe mental illness are dangerous more often than not.						
6	Health/social care staff know more about the lives of people treated for a mental illness than do family members or friends.						
7	If I had a mental illness, I would never admit this to my **colleagues** for fear of being treated differently.						
8	Being a health/social care professional in the area of mental health is **not** like being a real health/social care professional.						
9	If a senior colleague instructed me to treat people with a mental illness in a disrespectful manner, I would **not** follow their instructions.						

Mental Illness: Clinicians' Attitudes Scale MICA-2 © 2010. Health Service and Population Research Department, Institute of Psychiatry, King's College London.
We would like to thank Aliya Kassam for her major contribution to the development of this scale.
Contact: Professor Graham Thornicroft. Email: graham.thornicroft@kcl.ac.uk

Kassam A., Glozier N., Leese M., Henderson C., Thornicroft G. (2010) Development and responsiveness of a scale to measure clinicians' attitudes to people with mental illness (medical student version). Acta Psychiatrica Scandinavica 122(2), 153-161.

http://doi.org/10.47626/2237-6089-2021-0291

Page 2

Mental Illness: Clinicians' Attitudes Scale

MICA-4

Note to researchers distributing this scale: please only use after reading instructions in "Manual for Researchers".

Instructions: for each of questions 1-16, please respond by **ticking one box only**. Mental illness here refers to conditions for which an individual would be seen by a psychiatrist.

		Strongly agree	Agree	Somewhat agree	Somewhat disagree	Disagree	Strongly disagree
10	I feel as comfortable talking to a person with a mental illness as I do talking to a person with a physical illness.						
11	It is important that any health/social care professional supporting a person with a mental illness also ensures that their physical health is assessed.						
12	The public does **not** need to be protected from people with a severe mental illness.						
13	If a person with a mental illness complained of physical symptoms (such as chest pain) I would attribute it to their mental illness.						
14	General practitioners should **not** be expected to complete a thorough assessment for people with psychiatric symptoms because they can be referred to a psychiatrist.						
15	I would use the terms 'crazy', 'nutter', 'mad' etc. to describe to colleagues people with a mental illness who I have seen in my work.						
16	If a colleague told me they had a mental illness, I would still want to work with them.						

Thank you very much for your help.

Mental Illness: Clinicians' Attitudes Scale MICA-2 © 2010. Health Service and Population Research Department, Institute of Psychiatry, King's College London.
We would like to thank Aliya Kassam for her major contribution to the development of this scale.
Contact: Professor Graham Thornicroft. Email: graham.thornicroft@kcl.ac.uk

Kassam A., Glozier N., Leese M., Henderson C., Thornicroft G. (2010) Development and responsiveness of a scale to measure clinicians' attitudes to people with mental illness (medical student version). Acta Psychiatrica Scandinavica 122(2), 153-161.
