# Supplementary material for: Adaptation to Brazilian Portuguese and Latin-American Spanish and psychometric properties of the Mental Illness Clinicians’ Attitudes Scale (MICA v4)
Source: Trends Psychiatry Psychother. 2023 Mar 7;45:e20210291. doi: 10.47626/2237-6089-2021-0291 (PMC10164403; doi:10.47626/2237-6089-2021-0291)
Supplement: Supplementary file 2 [file 2238-0019-trends-45-e20210291-suppl2.pdf]

Supplementary Material S2

Página 1

Trastorno Mental: Escala de actitudes de los médicos. MICA

Nota a los investigadores que distribuyen esta escala: Por favor, utilice solo después de leer las instrucciones en el "Manual del Investigador".
Instrucciones: Por cada una de las preguntas del número 1 a la 16, por favor responda marcando una sola casilla. La enfermedad mental se refiere aquí a las condiciones por las cuales un individuo sería visto por un psiquiatra.

	Totalmente de acuerdo	De acuerdo	Algo de acuerdo	Algo en desacuerdo	En desacuerdo	Totalmente en desacuerdo
**1** Solo estudio sobre enfermedades mentales cuando tengo que hacerlo, y no me molestaría leer material adicional sobre esto.						
**2** Las personas con trastornos mentales graves nunca pueden recuperarse completamente para tener una buena calidad de vida.						
**3** Trabajar en el campo de la salud mental es tan respetable como trabajar en otros campos de la salud y de la atención social.						
**4** Si yo tuviera o padeciera una enfermedad mental nunca se lo haría saber a mis **amigos** porque tendría miedo de ser tratado de una forma diferente.						
**5** Las personas con trastornos mentales graves son más peligrosas que otras que no la tienen.						
**6** El personal de la salud y de asistencia social conocen más sobre la vida de las personas tratadas por una enfermedad mental que los miembros de su familia y amigos.						
**7** Si yo padeciera una enfermedad mental nunca lo admitiría a mis **colegas** porque tendría miedo de ser tratado de una forma diferente.						
**8** Ser un profesional de la asistencia social en la salud mental no es ser un verdadero profesional de la asistencia social en la salud.						
**9** Si un colega con mucha experiencia me instruye a tratar a las personas con una enfermedad mental de una manera irrespetuosa, no seguiría sus instrucciones.						

http://doi.org/10.47626/2237-6089-2021-0291

Página 2

Trastorno Mental: Escala de actitudes de los médicos. MICA

Nota a los investigadores que distribuyen esta escala: Por favor, utilice solo después de leer las instrucciones en el "Manual del Investigador".

Instrucciones: Por cada una de las preguntas del número 1 a la 16, por favor responda marcando una sola casilla. La enfermedad mental se refiere aquí a las condiciones por las cuales un individuo sería visto por un psiquiatra.

	Totalmente de acuerdo	De acuerdo	Algo de acuerdo	Algo en desacuerdo	En desacuerdo	Totalmente en desacuerdo
**10** Me siento cómodo lo mismo hablando con una persona con un trastorno mental como hablando con una persona con una enfermedad física.						
**11** Es importante que todo profesional de la salud y asistencia social cuidando de una persona con una enfermedad mental se asegure de que su salud física sea evaluada.						
**12** La población no necesita ser protegida de las personas con una enfermedad mental grave.						
**13** Si una persona con una enfermedad mental se queja de síntomas físicos (como dolor en el pecho) se lo atribuiría a su enfermedad mental.						
**14** Los médicos generales no deberían esperar a completar una evaluación exhaustiva de las personas con síntomas psiquiátricos porque pueden referirlos para un psiquiatra.						
**15** Yo usaría los términos "loco", "chiflado" etc. para describir con mis colegas a las personas con una enfermedad mental que veo (trato) en mi trabajo.						
**16** Si un colega me dice que tiene una enfermedad mental, me gustaría continuar trabajando con él.						

Mental Illness: Clinicians' Attitudes Scale MICA-2 © 2010.Health Service and Population Research Department, Institute of Psychiatry, King's College London.
We would like to thank Aliya Kassam for her major contribution to the development of this scale.
Contact: ProfessorGraham Thornicroft. Email: graham.thornicroft@kcl.ac.uk

Kassam A., Glozier N., Leese M., Henderson C., Thornicroft G. (2010) Development and responsiveness of a scale to measure clinicians' attitudes to people with mental illness (medical student version). Acta Psychiatrica Scandinavica 122(2), 153-161.
