# Supplementary material for: Adaptation to Brazilian Portuguese and Latin-American Spanish and psychometric properties of the Mental Illness Clinicians’ Attitudes Scale (MICA v4)
Source: Trends Psychiatry Psychother. 2023 Mar 7;45:e20210291. doi: 10.47626/2237-6089-2021-0291 (PMC10164403; doi:10.47626/2237-6089-2021-0291)
Supplement: Supplementary file 3 [file 2238-0019-trends-45-e20210291-suppl3.pdf]

http://doi.org/10.47626/2237-6089-2021-0291

Supplementary Material S3

Página 1

Doença Mental: Escala de atitudes dos médicos. **MICA - 4**

Nota dos investigadores que distribuem esta escala: utilize somente após ler as instruções no "Manual dos pesquisadores".

Instruções: para cada uma das perguntas de 1 a 16, por favor responda assinalando apenas uma alternativa. Doença mental aqui se refere às condições pelas quais um indivíduo poderia ser atendido por um psiquiatra.

	Concordo Plenamente	Concordo Parcialmente	Concordo	Discordo Parcialmente	Discordo	Discordo Plenamente
**1.** Só estudo sobre saúde mental quando necessito, mas não me incomodaria em ler material adicional sobre o assunto.						
**2.** Pessoas com quadro mental grave nunca podem se recuperar o suficiente para ter uma boa qualidade de vida.						
**3.** Trabalhar no campo da saúde mental é tão respeitável quanto em outros campos da saúde e da atenção social.						
**4.** Se eu tivesse uma doença mental nunca admitiria isso para meus amigos porque eu teria medo de ser tratado de modo diferente.						
**5.** Pessoas com transtorno mental grave são geralmente mais perigosos que os demais.						
**6.** Profissionais da saúde e do serviço social conhecem mais sobre a vida das pessoas tratadas por doença mental do que os membros da família ou amigos.						
**7.** Se eu tivesse uma doença mental eu nunca admitiria isso aos meus colegas de trabalho por medo de ser tratado de modo diferente.						
**8.** Ser um profissional da saúde ou do serviço social na área da saúde mental não é ser um verdadeiro profissional da saúde ou do serviço social.						
**9.** Se um colega mais experiente me orientar a tratar pessoas com doença mental de uma maneira desrespeitosa, eu não seguiria suas instruções.						

http://doi.org/10.47626/2237-6089-2021-0291

Doença Mental: Escala de atitudes dos médicos. MICA - 4

Nota dos investigadores que distribuem esta escala: utilize somente após ler as instruções no "Manual dos pesquisadores".

Instruções: para cada uma das perguntas de 1 a 16, por favor, responda assinalando apenas uma alternativa. Doença mental aqui se refere às condições pelas quais um indivíduo poderia ser atendido por um psiquiatra.

	Concordo Plenamente	Concordo Parcialmente	Concordo	Discordo Parcialmente	Discordo	Discordo Plenamente
**10.** Eu me sinto tão confortável em conversar com uma pessoa com doença mental quanto conversar com uma pessoa com uma doença física.						
**11.** É importante que qualquer profissional da saúde ou de assistência social quando der suporte a uma pessoa com uma doença mental também garanta que sua saúde física seja avaliada.						
**12.** A população não precisa ser protegida das pessoas com uma doença mental grave.						
**13.** Ser uma pessoa com uma doença mental se queixar de um sintoma físico (como uma dor no peito) eu atribuiria isso a sua doença mental.						
**14.** Clínicos gerais não deveriam esperar realizar uma avaliação mais completa de pessoas com sintomas psiquiátricos já que eles poderiam ser encaminhados a um psiquiatra.						
**15.** Eu usaria os termos "louco", "demente", "maluco" etc. para descrever para colegas de profissão as pessoas com doença mental que atendo no meu trabalho.						
**16.** Se um colega me conta que ele tem uma doença mental, eu seguiria trabalhando com ele.						

Mental Illness: Clinicians' Attitudes Scale MICA-2 © 2012 Health Service and Population Research Department, Institute of Psychiatry, King's College London.
We would like to thank Aliya Kassam for her major contribution to the development of this scale.
Contact: Professor Graham Thornicroft. Email: graham.thornicroft@kcl.ac.uk

Kassam A., Glozier N., Leese M., Henderson C., Thornicroft G. (2010) Development and responsiveness of a scale to measure clinicians' attitudes to people with mental illness (medical student version). Acta Psychiatrica Scandinavica 122(2), 153-161.
