# Supplementary material for: Adaptation to Brazilian Portuguese and Latin-American Spanish and psychometric properties of the Mental Illness Clinicians’ Attitudes Scale (MICA v4)
Source: Trends Psychiatry Psychother. 2023 Mar 7;45:e20210291. doi: 10.47626/2237-6089-2021-0291 (PMC10164403; doi:10.47626/2237-6089-2021-0291)
Supplement: Supplementary file 4 [file 2238-0019-trends-45-e20210291-suppl4.pdf]

http://doi.org/10.47626/2237-6089-2021-0291
Adaptation and psychometric properties of the Mental Illness Clinicians' Attitudes Scale (MICA v4) to Brazilian Portuguese and Latin-American Spanish - Vistorte et al.

Supplementary Material S4
Distribution of the MICA responses comparing Portuguese and Spanish samples

	Items	Strongly Agree	Agree	Somewhat Agree	Somewhat Disagree	Disagree	Strongly Disagree	P value (χ^2)
(1) I just learn about mental health when I have to, and would not bother reading additional material on it.	1 (Spanish Sample)	85(35.3%)	58(24.1%)	42(17.4%)	14(5.8%)	22(5.8%)	20(8.3%)	.53
	1 (Portuguese Sample)	57(39%)	30(20.5%)	20(13.7%)	17(11.6%)	12(8.2%)	10(6.8%)	
(2) People with a severe mental illness can never recover enough to have a good quality of life.	2 (Spanish Sample)	11(4.6%)	30(12.4%)	31(12.9%)	38(15.8%)	81(33.6%)	50(20.7%)	.21
	2 (Portuguese Sample)	5(3.4%)	9(6.2%)	7(4.8%)	20(13.7%)	55(37.7%)	50(34.2%)	
(3) Working in the mental health field is just as respectable as other fields of health and social care.	3 (Spanish Sample)	229(95%)	7(2.9%)	5(2.1%)	-	-	-	.27
	3 (Portuguese Sample)	129(88.4%)	3(2.1%)	10(6.8%)	2(1.4%)	-	2(1.4%)	
(4) If I had a mental illness, I would never admit this to my friends because I would fear being treated differently.	4 (Spanish Sample)	9(3.7%)	15(6.2%)	13(5.4%)	20(8.3%)	83(34.4%)	101(41.9%)	.85
	4 (Portuguese Sample)	3(2.1%)	13(8.9%)	13(5.4%)	22(15.1%)	43(29.5%)	59(40.4%)	
(5) People with a severe mental illness are dangerous more often than not.	5 (Spanish Sample)	19(7.9%)	41(17%)	44(18.3%)	47(19.5%)	57(23.7%)	33(13.7%)	.03
	5 (Portuguese Sample)	7(4.2%)	22(15.1%)	7(4.8%)	36(24.7%)	50(34.2%)	24(16.4%)	
(6) Health/social care staff know more about the lives of people treated for a mental illness than do family members or friends.	6 (Spanish Sample)	30(12.4%)	52(21.6%)	53(22%)	39(16.2%)	51(21.2%)	16(6.6%)	.16
	6 (Portuguese Sample)	12(8.2%)	39(26.7%)	21(14.4%)	39(26.7%)	23(15.8%)	12(8.2%)	
(7) If I had a mental illness, I would never admit this to my colleagues for fear of being treated differently.	7 (Spanish Sample)	9(3.7%)	15(6.2%)	22(9.1%)	25(10.4%)	94(39%)	76(31.5%)	.01
	7 (Portuguese Sample)	5(3.4%)	15(10.3%)	14(9.6%)	33(22.6%)	53(36.3%)	26(17.8%)	
(8) Being a health/social care professional in the area of mental health is not like being a real health/social care professional.	8 (Spanish Sample)	6(2.5%)	5(2.1%)	5(2.1%)	3(1.2%)	60(24.9%)	162(67.2%)	.31
	8 (Portuguese Sample)	3(2.1%)	-	4(2.7%)	5(3.4%)	29(19.9%)	105(71.9%)	
(9) If a senior colleague instructed me to treat people with a mental illness in a disrespectful manner, I would not follow their instructions.	9 (Spanish Sample)	187(77.6%)	14(5.8%)	11(4.6%)	4(1.7%)	7(2.9%)	18(7.5%)	.30
	9 (Portuguese Sample)	111(76%)	3(2.1%)	17(11.6%)	3(2.1%)	2(1.4%)	10(6.8%)	
(10) I feel as comfortable talking to a person with a mental illness as I do talking to a person with a physical illness.	10 (Spanish Sample)	123(51%)	63(26.1%)	23(9.5%)	7(2.9%)	23(9.5%)	2(0.8%)	.14
	10 (Portuguese Sample)	73(50%)	35(24%)	21(14.4%)	9(6.2%)	7(4.8%)	1(0.7%)	
(11) It is important that any health/social care professional supporting a person with a mental illness also ensures that their physical health is assessed.	11 (Spanish Sample)	190(78.8%)	36(14.9%)	11(4.6%)	2(0.8%)	-	2(0.8%)	.01
	11 (Portuguese Sample)	122(83.6%)	3(2.1%)	16(11%)	2(1.4%)	2(1.4%)	1(0.7%)	
(12) The public does not need to be protected from people with a severe mental illness.	12 (Spanish Sample)	60(24.9%)	48(19.9%)	31(12.9%)	63(26.1%)	28(11.6%)	11(4.6%)	.43
	12 (Portuguese Sample)	28(19.2%)	39(26.7%)	19(13%)	36(24.7%)	19(13%)	5(3.4%)	
(13) If a person with a mental illness complained of physical symptoms (such as chest pain) I would attribute it to their mental illness.	13 (Spanish Sample)	2(0.8%)	6(2.5%)	10(4.1%)	36(14.9%)	89(36.9%)	98(40.7%)	.02
	13 (Portuguese Sample)	-	7(4.8%)	1(0.7%)	31(21.2%)	61(41.8%)	46(31.5%)	
(14) General practitioners should not be expected to complete a thorough assessment for people with psychiatric symptoms because they can be referred to a psychiatrist.	14 (Spanish Sample)	15(6.2%)	11(4.6%)	20(8.3%)	35(14.5%)	88(36.5%)	72(29.9%)	.16
	14 (Portuguese Sample)	4(2.7%)	14(9.6%)	5(3.4%)	28(19.2%)	44(30.1%)	51(34.9%)	
(15) I would use the terms 'crazy', 'nutter', 'mad' etc. to describe to colleagues people with a mental illness who I have seen in my work.	15 (Spanish Sample)	1(0.4%)	1(0.4%)	1(0.4%)	2(0.8%)	47(19.5%)	189(78.4%)	.25
	15 (Portuguese Sample)	1(0.7%)	3(2.1%)	2(1.4%)	8(5.5%)	30(20.5%)	102(69.9%)	
(16) If a colleague told me they had a mental illness, I would still want to work with them.	16 (Spanish Sample)	135(56%)	75(31.1%)	24(10%)	2(0.8%)	2(0.8%)	3(1.2%)	.01
	16 (Portuguese Sample)	97(66.4%)	12(8.2%)	36(24.7%)	1(0.7%)	-	-	
